# FIELD: A comprehensive FarmIng Electrical LoaD measurements dataset from 30 three-phase dairy farms in Germany

**DOI:** 10.1038/s41597-025-06094-2

**Published:** 2025-11-19

**Authors:** Apostolos Vavouris, Lina Stankovic, Vladimir Stankovic, Jiufeng Shi

**Affiliations:** 1 Department of Electronic and Electrical Engineering, University of Strathclyde, Glasgow, G1 1XQ United Kingdom; 2 Data Science Discovergy GmbH, Heidelberg, Germany

**Keywords:** Energy modelling, Electrical and electronic engineering

## Abstract

The industrial sector, which is characterised by complex and diverse equipment and processes, is a core contributor to carbon emissions. Agriculture, in particular, has seen an increase in carbon dioxide emissions, in contrast to methane and nitrous oxide emissions, due to the increased use of novel, energy-intensive agricultural technology. Therefore, to meet Net Zero targets, analyses of the energy consumption of dairy technologies are necessary to enhance the energy efficiency of the agricultural equipment. Incidentally, the only public electrical measurements datasets of agricultural technology are one for poultry feed for 111 days, one for a synthetic hourly load profile of a dairy farm in Finland and another with aggregate readings and submetered milking robots from three farms in Germany. This dataset aims to accelerate energy efficiency and management services in the sector by curating and providing access to a comprehensive, first-of-its-kind, granular (1-sec) power readings dataset from 30 dairy farms across Germany with 72 submetered energy-intensive equipment, for a period of over one year.

## Background & Summary

For the first time in the history of COP conferences, in COP28, sustainable agriculture was recognised as a path to achieve the long-term goals of the Paris Agreement (https://www.cop28.com/en/food-and-agriculture). According to the International Energy Agency, in 2020, the agricultural sector produced 0.4 GtCO_2_eq, with an expected increase in emissions by 20%, by 2050 under the Stated Policies Scenario, which is much higher than the Sustainable Development Scenario — with an expected decrease of carbon dioxide (CO_2_) emissions by 60% — and the Net Zero Emissions by 2050 Scenario — with an expected decrease of the CO_2_ emissions by 100%^1^. Indeed, a recent report on agricultural emissions shows that although methane (CH_4_) and nitrous oxide (N_2_O) emissions in the UK have been on a steady decrease since 1990, CO_2_ emissions have increased by 22%^2^, due to the increasing use of energy-intensive agricultural technologies.

In the agricultural technologies sector, diverse and complex energy-intensive equipment has not been explored, creating a barrier in the development of advanced energy efficiency and management services for the sector to mitigate CO_2_ emissions. Further to that, CO_2_ emissions from UK agriculture remain a concern, totalling 5.5 MtCO_2_e, nearly 2% of the country’s total carbon emissions^2^. This represents a 22% increase since 1990, largely driven by the growing adoption of energy-intensive agricultural technologies. Indeed, according to the Agriculture and Horticulture Development Board (https://ahdb.org.uk), the increasing electricity requirements of dairy farms are highlighted together with potential savings of up to 12% in electricity bills through the monitoring and analysis of energy usage. Efforts on understanding and quantifying energy consumption in dairy farms have focused on monitoring, predictive modelling of energy consumption patterns^3^, or simulating^4^ energy consumption rather than metering and quantifying the actual consumption of dairy equipment. Although the potential to accelerate the decarbonisation of the dairy sector through big data, artificial intelligence (AI), and real-time sensor monitoring was recently highlighted^5^, the lack of detailed energy measurement datasets creates a barrier in the development of such data-driven analyses. In fact, a recent study on load disaggregation for dairy farms^6^ demonstrated that due to the lack of detailed labelled energy consumption datasets and due to the large differences between diverse dairy equipment, and the different naming conventions of individual equipment during monitoring, transfer learning of Non-Intrusive Load Monitoring (NILM) models across different dairy settings was limited.

In the agricultural sector, publicly available energy use datasets are available only for a poultry feed facility in Brazil^7^ – comprising active, reactive, apparent, voltage and current measured for 111 days at 1-sec granularity – and 2 sites for dairy farms. An hourly synthetic aggregate electrical consumption and estimated PV production data for a year were released in^8^ without any measurements for a small-medium dairy farm in Finland to explore integration of renewables in a microgrid. More recently, aggregate energy consumption measurements of three monitored dairy farms in Germany with nine submetered points related to milking robots sampled at 10 seconds for a year were released in^9^. Though this dataset gave insights into the load profiles of voluntary milking systems, there are more complex loads in the dairy sector that remain undocumented.

This paper describes the curation of and release of FIELD^10^, a comprehensive electrical loads measurement dataset for a diverse range of typical energy-intensive activities, including detailed labelling and load characteristics information that improves the understanding of the diverse dairy farming activities. The dataset contains granular 1-second active power, aggregated and sub-metered, three-phase readings from 30 dairy farms for a period spanning over 1 year (1st of February 2020 – 5th of March 2021) that enables seasonal variation analyses in addition to activity recognition, energy consumption analysis of individual energy intensive activities, automated load shifting and demand response, renewables and energy storage integration. To the best of our knowledge, and at the time of publication, this dataset is the largest and only electricity dataset providing aggregate load consumption readings from 30 monitored farms together with a range of submetered readings for a diverse range of energy intensive dairy equipment, spanning voluntary milking systems (milking robots), traditional milking parlours and their submetered components, diverse feeding equipment, cleaning, ventilating, lighting, heat exchanging, and other farming technologies. The dataset has been used as an enabler for the development of a co-created methodology based on a complex mixed-methods approach, akin to prior work^11^, for the largely non-standardised dairy industry. The value of the FIELD dataset for training, through transfer learning, of a NILM-enabled load scheduler has been demonstrated under low- and very-low frequency data scenarios^12^.

The FIELD dataset supports key research areas, including the development of NILM algorithms tailored to the heterogeneous and non-standardised nature of dairy farm equipment, energy disaggregation and profiling, and predictive maintenance of agricultural machinery. Furthermore, the dataset supports research into demand-side management strategies such as automated load scheduling, load shifting, and integration of renewables and storage systems in agri-settings. For stakeholders, including policymakers, energy consultants, and farm operators, FIELD provides an empirical foundation to inform energy efficiency policies, benchmark energy performance, and design targeted interventions to reduce carbon emissions. The dataset also enables technology developers to design energy management systems that are better aligned with the operational practices and constraints of dairy farms. Overall, FIELD contributes to bridging the data gap in agricultural energy research, facilitating evidence-based decision-making and accelerating the decarbonisation of the agricultural sector.

## Methods

Dairy farms, included in the dataset, were sampled across Germany with the smart metering and sub-metering infrastructure installed through the utility provider. The smart metering hardware complied with the smart metering standards as published by CENELEC^13^. A total of 31 dairy farms were monitored, containing a wide variety of novel dairy technologies, including voluntary milking robots, automated scrapers and climate-control barn ventilation. Out of these 31 farms, due to connectivity and data transmission issues, aggregate data could not be collected from the farms with the following identification (id) numbers: 8, 13, 14, and 19, while submetered data were collected from all the farms, with the number of monitored dairy equipment ranging from 1 to 4 metering points per dairy farm. Table 1 presents a summary of the data available, including the monitored duration and the availability of aggregate and/or submetering readings.

**Table 1:** Overview of measurements available per farm.

ID	From	To	Period	Aggregate	Submetering
**1**	14/03/2020 09:26:21	05/03/2021 00:00:00	11mo 18d 14h 33m 39s	*✓*	*✓*(4)
**2**	13/02/2020 09:09:55	09/07/2020 12:54:48	4mo 26d 3h 44m 53s	*✓*	*✓*(4)
**3**	01/02/2020 00:00:00	05/03/2021 00:00:00	1y 1mo 4d	*✓*	*✓*(3)
**4**	05/02/2020 09:14:26	05/03/2021 00:00:00	1y 27d 14h 45m 34s	*✓*	*✓*(4)
**5**	01/02/2020 00:00:00	05/03/2021 00:00:00	1y 1mo 4d	*✓*	*✓*(4)
**6**	03/02/2020 13:37:20	05/03/2021 00:00:00	1y 1mo 1d 10h 22m 40s	*✓*	*✓*(1)
**7**	03/02/2020 12:58:33	05/03/2021 00:00:00	1y 1mo 1d 11h 1m 27s	*✓*	*✓*(1)
**8**	03/02/2020 12:58:51	05/03/2021 00:00:00	1y 1mo 1d 11h 1m 9s	✗	*✓*(1)
**9**	13/02/2020 09:06:09	03/09/2020 14:55:35	6mo 21d 5h 49m 26s	*✓*	*✓*(4)
**10**	09/03/2020 12:27:26	05/03/2021 00:00:00	11mo 23d 11h 32m 34s	*✓*	*✓*(3)
**11**	26/02/2020 11:26:25	27/08/2020 08:35:16	6mo 21h 8m 51s	*✓*	*✓*(4)
**12**	26/02/2020 11:26:27	03/09/2020 14:55:35	6mo 8d 3h 29m 8s	*✓*	*✓*(3)
**13**	11/03/2020 06:59:40	05/03/2021 00:00:00	11mo 21d 17h 0m 20s	✗	*✓*(1)
**14**	11/03/2020 06:07:47	05/03/2021 00:00:00	11mo 21d 17h 52m 13s	✗	*✓*(1)
**15**	11/03/2020 06:21:30	02/03/2021 13:41:44	11mo 19d 7h 20m 14s	✗(^*^)	✗(^*^)
**16**	11/03/2020 06:17:03	05/03/2021 00:00:00	11mo 21d 17h 42m 57s	*✓*	*✓*(2)
**17**	14/03/2020 09:47:38	05/03/2021 00:00:00	11mo 18d 14h 12m 22s	*✓*	*✓*(2)
**18**	14/03/2020 09:35:21	05/03/2021 00:00:00	11mo 18d 14h 24m 39s	*✓*	*✓*(4)
**19**	14/03/2020 09:36:38	05/03/2021 00:00:00	11mo 18d 14h 23m 22s	✗	*✓*(1)
**20**	16/04/2020 10:31:24	05/03/2021 00:00:00	10mo 16d 13h 28m 36s	*✓*	*✓*(1)
**21**	16/04/2020 10:44:02	01/02/2021 09:10:23	9mo 15d 22h 26m 21s	*✓*	*✓*(4)
**22**	13/02/2020 09:09:54	03/09/2020 14:55:36	6mo 21d 5h 45m 42s	*✓*	*✓*(1)
**23**	16/04/2020 10:53:50	05/03/2021 00:00:00	10mo 16d 13h 6m 10s	*✓*	*✓*(4)
**24**	11/03/2020 06:19:54	05/03/2021 00:00:00	11mo 21d 17h 40m 6s	*✓*	*✓*(4)
**25**	10/02/2020 13:36:26	20/10/2020 10:02:27	8mo 9d 20h 26m 1s	*✓*	*✓*(1)
**26**	18/02/2020 12:51:12	05/03/2021 00:00:00	1y 14d 11h 8m 48s	*✓*	*✓*(1)
**27**	10/02/2020 13:35:29	05/03/2021 00:00:00	1y 22d 10h 24m 31s	*✓*	*✓*(3)
**28**	10/02/2020 13:31:46	14/08/2020 01:26:57	6mo 3d 11h 55m 11s	*✓*	*✓*(2)
**29**	18/02/2020 12:35:29	05/03/2021 00:00:00	1y 14d 11h 24m 31s	*✓*	*✓*(2)
**30**	18/02/2020 12:52:53	05/03/2021 00:00:00	1y 14d 11h 7m 7s	*✓*	*✓*(1)
**31**	18/02/2020 12:53:19	05/03/2021 00:00:00	1y 14d 11h 6m 41s	*✓*	*✓*(1)
	**01/02/2020 00:00:00**	**05/03/2021 00:00:00**	**1y 1mo 4d**	**26**	**72**

The submetering column shows the number of submetered dairy equipment. ^*^No activity was identified in the measurements from farm 15, and therefore, the electrical measurements are not included in the dataset.

### Aggregate readings

Each of the three phases, with a 380V connection, is monitored individually per farm. Three active power level readings were collected and transmitted every 1 second, corresponding to each of the three installation phases. In case of transmission failure within a given time period, all three phase readings during that period are lost.

### Submetered equipment

On each dairy farm, up to 4 different points were selected for power monitoring that corresponded to different energy-intensive activities. The monitored dairy equipment can be attributed to the following activities:

1. milking: including milking robots, breast and vacuum pumps
2. feeding: including grist mills, feeding augers and feeding cabinets
3. cleaning: including manure removers, heavy duty cleaners, pipe flushers, dungings and pressure water cleaners
4. ventilation: including cowshed fans, barn fans and ventilation compartments
5. lighting: including indoor and outdoor lighting and infrared lighting
6. heating & cooling: including heating plates, warming cabinets and compressors
7. miscellaneous: including straw barns for the feeding of the animals, circulation pumps, industrial washing/ironing machines required for the cleaning/disinfection of the farmers’ clothes, gas cannons to scare pests and equipment available in farms’ kitchens such as salamanders and extraction hoods

**Table 2:** Summary of the submetered points (*SP*) per farm.

ID	*SP*_1_	*SP*_2_	*SP*_3_	*SP*_4_
**1**	Straw barn	Grist mill 1	Grist mill 2	Manure removal
**2**	Unassigned	Light path 1	*ϕ*_1,2_: light path 2	Gang farrowing
**3**	Heavy duty cleaner	Lighting	Night/outdoor lights	—
**4**	Unassigned	Lighting	Fan cowshed	*ϕ*_1_: lights stable
				*ϕ*_2_: office
				*ϕ*_3_: lights hall
**5**	Unassigned	Climate computer circulation pump	*ϕ*_1_: distribution	Ventilation
			*ϕ*_2_: alarm	
			*ϕ*_3_: ventilation	
**6**	Lights	—	—	—
**7**	Climate	—	—	—
**8**	Infrared lights	—	—	—
**9**	Heating plates 1	Heating plates 2	*ϕ*_2,3_: infrared lights 1:	*ϕ*_1_: infrared lights 2
**10**	32A power socket	Feeding	Slurry	—
**11**	Fan 1	Fan 2	Fan 3	Fan 4
**12**	Feed auger 1	Feed auger 2	Metal halide lamps	—
**13**	Sauna	—	—	—
**14**	Washing machine	—	—	—
**16**	*ϕ*_1_: Freezer cellar	*ϕ*_1_: lights	—	—
	*ϕ*_2_: warming cabinet	*ϕ*_2_: sliding car door	—	—
	*ϕ*_3_: thermal bridge	*ϕ*_3_: extractor hood	—	—
**17**	Slider front	*ϕ*_2,3_: slider rear	—	—
**18**	Pressure water cleaning	Vacuum pump	Compressor	Milking robot
**19**	Feeding auger	—	—	—
**20**	Milking robot	—	—	—
**21**	Water treatment	Pipe cooler pump	Water pump	*ϕ*_1,2_: dunging
**22**	Feeding cabinet	—	—	—
**23**	Breast pump	Vacuum pump	Pipe flusher	Fan barn
**24**	Fryer 1	Fryer 2	Ironing machine	Salamander
**25**	Power socket	—	—	—
**26**	Lights & feeding	—	—	—
**27**	Three-phase 380V power socket	*ϕ*_1,2_: ventilation 1	Ventilation 2 & unassigned	—
**28**	Ventilation 1	*ϕ*_1,2_: ventilation 2	—	—
		*ϕ*_3_: gas cannon	—	—
**29**	Lighting	Feeding	—	—
**30**	Infrared lighting	—	—	—
**31**	Lighting	—	—	—

Equipment that is not connected to all three phases but either one or two, is marked with *ϕ*_*i*_, where *i* = 1, 2, 3 are the connected phases.

**Table 3:** Summary of the maximum power levels of each submetered equipment per farm and per phase.

ID	*SP*_1_ [W]	*SP*_2_ [W]	*SP*_3_ [W]	*SP*_4_ [W]
**1**	3,933	19	2,507	4,509
**2**	4,945	9,268	*ϕ*_1,2_: 2,681	29,253
**3**	14,679	5,251	1,290	—
**4**	16	2,443	13,510	*ϕ*_1_: 7,808
				*ϕ*_2_: 3,580
				*ϕ*_3_: 5,700
**5**	15	119	*ϕ*_1_: 70	7,823
			*ϕ*_2_: 24	
			*ϕ*_3_: 1,409	
**6**	8,358	—	—	—
**7**	9,173	—	—	—
**8**	8,293	—	—	—
**9**	8,088	6,852	*ϕ*_2,3_: 4,738	*ϕ*_1_: 2,198
**10**	5,426	17,838	22,330	—
**11**	3,365	3,425	3,433	3,530
**12**	2,196	2,222	6,545	—
**13**	18,530	—	—	—
**14**	17,242	—	—	—
**16**	*ϕ*_1_: 8,377	*ϕ*_1_: 1,416	—	—
	*ϕ*_2_: 2,305	*ϕ*_2_: 249	—	—
	*ϕ*_3_: 1,264	*ϕ*_3_: 7,221	—	—
**17**	571	*ϕ*_2,3_: 2,252	—	—
**18**	3,362	10,310	4,536	3,966
**19**	4,846	—	—	—
**20**	6,541	—	—	—
**21**	7,658	5,571	4,282	*ϕ*_1,2_: 1,378
**22**	24,967	—	—	—
**23**	2,146	12,037	29,391	4,724
**24**	23,370	6,267	12,225	202
**25**	19,650	—	—	—
**26**	28,492	—	—	—
**27**	10,346	*ϕ*_1,2_: 478	1,283	—
**28**	4,052	*ϕ*_1,2_: 2,947	—	—
		*ϕ*_3_: 736	—	—
**29**	21,774	12,058	—	—
**30**	16,202	—	—	—
**31**	2,618	—	—	—

Specifically, the submetering points (*SPs*) and the respective equipment that were monitored on each farm (note that all monitored equipment are three-phase powered unless stated otherwise) are presented in Tables 2 and 3.

**Fig. 1:**
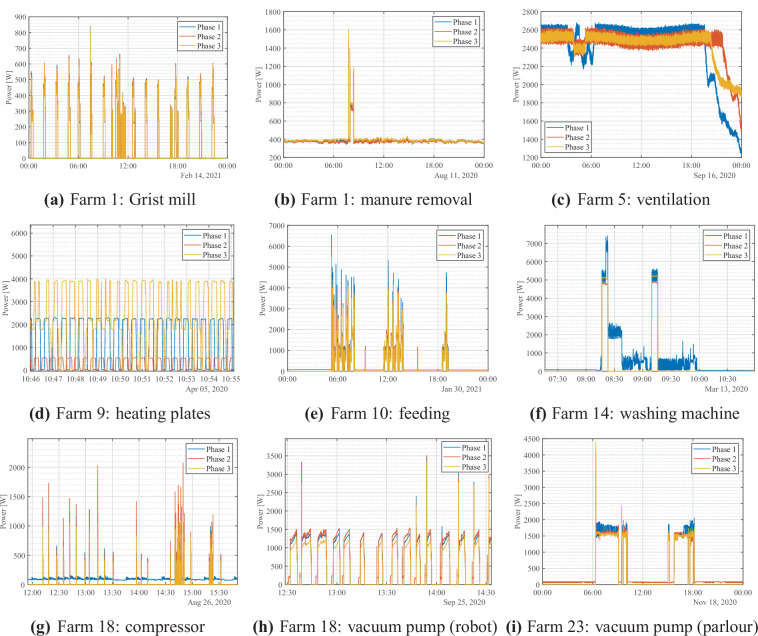
A range of selected submetered equipment.

A selection of the load profiles of different monitored equipment is included in Fig. 1, where the per-phase consumption of the monitored equipment is presented. Through the phase-by-phase presentation of the load consumption, the level of load balancing of each individual equipment can be observed. The selected equipment include a wide variety of farming activities such as milking (see Fig. 1h,i), feeding (see Fig. 1a,e), cleaning (see Fig. 1b), ventilation (see Fig. 1c), heating & cooling (see Fig. 1d,g) and miscellaneous activities (see Fig. 1f). A 24-hour period is presented for all the monitored equipment, apart from equipment that have sparse activations — i.e., industrial washing machine, see Fig. 1f — where a single activation is presented, and equipment with dense repetitive consumption patterns — i.e., heating plates (see Fig. 1d), compressor (see Fig. 1g), and milking robot vacuum pump (see Fig. 1h) — where for illustration purposes, load profiles ranging from several minutes to a few hours is presented.

As this dataset aims to enable the comprehensive study of different farming equipment, understanding of the underlying farming processes is essential for the effective application of this dataset to other dairy farms via transfer learning, for example. It is important to note the fundamental difference between the two key milking approaches: milking using a parlour (either a traditional herringbone or a rotary) and milking using voluntary robot milking machines. In this dataset, both types of milking processes have been monitored, with Farms 18, 20 and 21 having voluntary milking robots installed, whereas Farm 23 has a parlour. On the farms with milking robots, the energy consumption of this equipment has been monitored both at the aggregate level as well as per sub-process of the milking robot, including water treatment, pipe cooler pump, water pump and dunging (see Table 2). The difference between the two milking approaches is evident by comparing Fig. 1h,i where in the farm with the voluntary milking robot, the vacuum pump is being used intermittently for a short period every time a cow approaches a milking robot to get milked, whereas in the case of a parlour, where multiple cows are milked simultaneously, there are two distinctive milking rounds (one in the morning and one in the evening) with the milking equipment running constantly during that time. Therefore, although the two equipment may perform a similar task, their consumption patterns vary significantly depending on the underlying farming method. Lastly, due to the variety in sizes across different farms, the same equipment may differ between the two farms (both in terms of power level and activation duration due to the different number of animals. It is worth noting, though, that the load consumption signal shape is similar, but the magnitude differs. This is also evident in the case of the industrial washing machine (see Fig. 1f), where although the maximum power level exceeds 17000 W (see Table 2), the pattern of the signal resembles those of residential washing machines as present in various domestic electrical datasets.

### Per-phase signal recovery

Both aggregate and submetered readings in the raw dataset were in the following format: *phase*_2_ = *phase*_2_ + *phase*_1_ and *phase*_3_ = *phase*_3_ + *phase*_2_ + *phase*_1_, where *phase*_*i*_ is the power level on phase *i* for *i* = 1, 2, 3. Thus, in order to recover per-phase power readings at each sample point, Algorithm 1 was adopted. Algorithm 1 recovers the original per phase aggregate reading from the aggregate collected per-phase vectors of each farm ${\overrightarrow{p}}_{id,1},{\overrightarrow{p}}_{id,2},{\overrightarrow{p}}_{id,3}$ with *id* = 1, . . . , 31, and 1, 2, and 3, correspond to Phase 1, 2 and 3, respectively.

#### Algorithm 1

Aggregate signal recovery.

As the submetered points in each farm were organised by distribution boards, with the submetered points on each distribution board numbered in ascending order, each submetered point time-series data contained the sum of the active power readings from the current point and all the submetered points that are connected to the same distribution point with an id smaller than the current one. Therefore, in order to recover the equipment-level per-phase signal, Algorithm 2 was adopted. Let ${\overrightarrow{p}}_{id,j,i,phase}$ be the collected per-phase power vector of the submetered point *i* of a distribution board *j*. The input to Algorithm 2 is the set of collected per-phase vectors of the submetered points (sp) on a distribution board (db), given by:

$${\overrightarrow{p}}_{id,db,1}=\left\{\begin{array}{l}{\overrightarrow{p}}_{id,db,1,1}\\ {\overrightarrow{p}}_{id,db,2,1}\\ \ldots \\ {\overrightarrow{p}}_{id,db,sp,1}\end{array}\right.,{\overrightarrow{p}}_{id,db,2}=\left\{\begin{array}{l}{\overrightarrow{p}}_{id,db,1,2}\\ {\overrightarrow{p}}_{id,db,2,2}\\ \ldots \\ {\overrightarrow{p}}_{id,db,sp,2}\end{array}\right.,\mathrm{and}\,{\overrightarrow{p}}_{id,db,3}=\left\{\begin{array}{l}{\overrightarrow{p}}_{id,db,1,3}\\ {\overrightarrow{p}}_{id,db,2,3}\\ .\ldots \\ {\overrightarrow{p}}_{id,db,sp,3}\end{array}\right.$$

where *sp* is the number of the submetered points on the specific db, and the outputs are the recovered per-phase readings for each submetered point per distribution board.

#### Algorithm 2

Submetered signal recovery

## Data Records

**Fig. 2:**
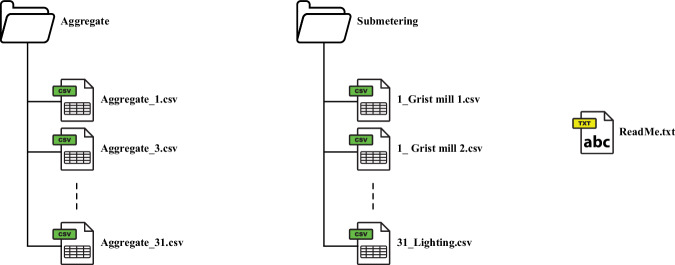
Dataset structure.

In line with the FAIR principles^14^, the UK Data Service’s recommended file formats for data sharing, reuse, and preservation^15^, and established practices in NILM literature^16^, the data are provided as comma-separated values (CSV) and text (TXT) files. For each farm where aggregated data are available (see Table 1), there is a single CSV file associated with the farm that contains the aggregated data readings. For each farm for which submetered data are available (see Table 1), there is a CSV file associated with each submetered dairy equipment that contains the submetered readings. Fig. 2 represents the structure of the dataset. The columns of the CSV files (“Aggregate_*id*.csv”) containing the aggregate data of each farm “*id*” are described in Table 4.

**Table 4:** Description of aggregate (“Aggregate_#.csv”).

Feature	Type	Description
**Time**	DateTime	The timestamp of the collected data in UTC [YYYY-MM-DD HH:mm:ss]
**phase1**	Float	The aggregated active power level on phase 1 in Watts [W]
**phase2**	Float	The aggregated active power level on phase 2 in Watts [W]
**phase3**	Float	The aggregated active power level on phase 3 in Watts [W]

The columns of the CSV files (“*id*_<<*equipment*>>.csv”) containing the submetered equipment data of each farm “*id*” are described in Table 5.

**Table 5:** Description of submetering (“#_ << *equipment* >>.csv”).

Feature	Type	Description
**Time**	DateTime	The timestamp of the collected data in UTC [YYYY-MM-DD HH:mm:ss]
**phase1**	Float	The submetered active power level on phase 1 in Watts [W]
**phase2**	Float	The submetered active power level on phase 2 in Watts [W]
**phase3**	Float	The submetered active power level on phase 3 in Watts [W]

A single ReadMe TXT file is included to provide additional information about the structure of the dataset. The format of the ReadMe file is the following:

- Dataset description
- Licensing
- Naming conventions
- File formats
- Farming equipment per site

The post-processed FIELD dataset can be accessed through the University of Strathclyde’s PURE data repository^10^.

## Technical Validation

Following on from the Methods section, a summary of the aggregate and submetered data availability across the 30 farms comparing the actual and the expected number of samples is presented in Table 6. Please note that at each time point, three active power readings, one from each of the three phases, were collected (both for aggregated and submetered samples).

**Table 6:** Data availability.

ID	Aggregate samples			Submetering samples		
	Expected	Available	[%]	Expected	Available	[%]
**1**	92,173,260	92,062,074	99.88	368,693,040	368,248,296	99.88
**2**	38,142,882	35,641,851	93.44	152,571,528	142,567,404	93.44
**3**	103,161,603	102,856,554	99.70	309,484,809	308,569,662	99.70
**4**	102,025,005	101,562,177	99.55	408,100,020	406,248,708	99.55
**5**	103,161,603	91,050,837	88.26	412,646,412	364,203,348	88.26
**6**	102,496,083	102,496,083	100.00	102,496,083	102,496,083	100.00
**7**	102,503,064	102,503,064	100.00	102,503,064	102,503,064	100.00
**8**	—	—	—	102,503,010	102,503,010	100.00
**9**	52,680,501	50,179,728	95.25	210,722,004	200,718,912	95.25
**10**	93,436,665	93,436,665	100.00	280,309,995	280,309,995	100.00
**11**	47,402,796	47,402,796	100.00	189,611,184	189,611,184	100.00
**12**	49,285,647	49,205,520	99.84	147,856,941	147,616,560	99.84
**13**	—	—	—	92,977,263	92,977,263	100.00
**14**	—	—	—	92,986,602	92,986,602	100.00
**16**	92,984,934	92,984,934	100.00	185,969,868	185,969,868	100.00
**17**	92,169,429	92,169,429	100.00	184,338,858	184,338,858	100.00
**18**	92,171,640	92,171,640	100.00	368,686,560	368,686,560	100.00
**19**	—	—	—	92,171,409	92,171,409	100.00
**20**	83,607,951	81,692,028	97.71	83,607,951	81,692,028	97.71
**21**	75,410,346	55,095,387	73.06	301,641,384	220,381,548	73.06
**22**	52,679,829	50,179,062	95.25	52,679,829	50,179,062	95.25
**23**	83,603,913	81,667,818	97.68	334,415,652	326,671,272	97.68
**24**	92,984,421	92,984,421	100.00	371,937,684	371,937,684	100.00
**25**	65,539,086	65,539,086	100.00	65,539,086	65,539,086	100.00
**26**	98,616,387	98,616,387	100.00	98,616,387	98,616,387	100.00
**27**	100,682,016	100,682,016	100.00	302,046,048	302,046,048	100.00
**28**	48,080,736	9,942,039	20.68	96,161,472	19,884,078	20.68
**29**	98,619,216	98,619,216	100.00	197,238,432	197,238,432	100.00
**30**	98,616,084	78,697,698	79.80	98,616,084	78,697,698	79.80
**31**	98,616,006	98,616,006	100.00	98,616,006	98,616,006	100.00
**Total**	**2,160,851,103**	**2,058,054,516**	**95.24**	**5,905,744,665**	**5,644,226,115**	**95.57**

Table 7 highlights large gaps due to metering infrastructure disconnection or due to meter malfunctioning. The majority of the metering disconnections lasted from a few hours to a couple of days. Farm 28 contains a notable gap that spans over 4 months — which also affects the data availability (see Table 6). Although only 20.68% of the timestamps were collected on this farm, the farm was not excluded from the dataset as it contained equipment that was not monitored in other farms. The identified gaps in Table 7 were left unfilled with NaN values included in the dataset. These large gaps could be filled through interpolation of average historical consumption data, but this step should be decided based on the use of the specific dataset. All the collected data streams were manually inspected (visual inspection) by an energy expert to assess the validity of the data. Negative values that were produced due to rounding errors, due to the post-processing of the metered data with absolute values ranging from E-14 to E-15 Watts, were set to zero. No erroneous spikes were identified in the collected energy readings. Spikes observed during the starting of equipment (especially inductive motors, such as compressors and pumps) that are the result of the inrush currents were not removed and are included in the dataset. Farms with only submetered loads and no aggregate (i.e., farms with IDs 8, 13, 14, and 19) were manually inspected and compared with similar loads from the dataset. As farm 13 and 14 contain unique submetered equipment that is not available on other farms, these could not be cross-validated through other farms in the dataset. The industrial scale washing machine load signature of farm 14 was compared with similar residential washing machines from publicly available datasets^17^. Although the industrial washing machine present in Farm 14 had a much higher power level, with the water heating element distributed across the three phases, the load pattern matched the residential ones. Lastly, the sum of the submetered loads was compared with the aggregate load on a per-phase basis to ensure that the total submetered power is less than or equal to the aggregate power at each sampling point.

**Table 7:** Data gaps.

ID	Start	Stop	Duration
**1**	21/01/2021 04:00:30	21/01/2021 13:02:50	9h 2m 20s
**2**	13/06/2020 16:32:25	23/06/2020 08:05:55	9d 15h 33m 30s
**3**	17/02/2020 07:37:51	18/02/2020 11:52:33	1d 4h 14m 42s
**4**	03/09/2020 14:55:36	05/09/2020 09:44:04	1d 18h 48m 28s
**5**	16/12/2020 16:10:04	01/02/2021 08:34:39	1mo 15d 16h 24m 35s
**9**	13/06/2020 16:32:24	13/06/2020 08:05:34	9d 15h 33m 10s
**12**	03/07/2020 20:01:06	04/07/2020 03:26:14	7h 25m 8s
**20**	13/06/2020 16:58:55	17/06/2020 05:11:47	3d 12h 12m 52s
	31/12/2020 10:50:40	04/01/2021 08:01:47	3d 21h 11m 7s
**21**	13/06/2020 16:58:54	17/06/2020 05:11:48	3d 12h 12m 54s
	09/07/2020 12:54:50	18/09/2020 12:31:35	2mo 8d 23h 36m 45s
	31/12/2020 10:50:41	04/01/2021 08:01:52	3d 21h 11m 11s
**22**	13/06/2020 16:32:24	23/06/2020 08:05:32	9d 15h 33m 8s
**23**	13/06/2020 16:58:55	17/06/2020 05:11:53	3d 12h 12m 58s
	31/12/2020 10:50:41	04/01/2021 08:01:54	3d 21h 11m 13s
**27**	20/07/2020 19:29:43	17/09/2020 12:02:35	1mo 27d 32m 52s
**28**	20/02/2020 15:39:38	22/06/2020 18:54:12	4mo 2d 3h 14m 34s
	02/07/2020 09:13:54	03/07/2020 14:17:21	1d 5h 3m 27s
	20/07/2020 19:29:44	12/08/2020 14:33:19	22d 19h 3m 35s
**30**	29/07/2020 10:37:04	31/07/2020 07:44:22	1d 21h 7m 18s
	06/08/2020 11:52:26	20/10/2020 11:02:48	2mo 13d 23h 10m 22s

## Usage Notes

The dataset is made available in CSV format, which can be easily accessed by the majority of the scientific computing packages, including MATLAB, SPSS, R and Python. The included README file explains the structure of each individual CSV file, including its contents and any known issues.

## Data Availability

The code to prepare, identify gaps, visualise and store the data was developed using MATLAB R2024a and deployed on a Windows machine. The algorithm used to post-process the data is presented in Methods Section.
